# Exploring Environmental Health on Weibo: A Textual Analysis of Framing Haze-Related Stories on Chinese Social Media

**DOI:** 10.3390/ijerph16132374

**Published:** 2019-07-04

**Authors:** Fan Yang, Jessica Wendorf Muhamad, Qinghua Yang

**Affiliations:** 1 Department of Communication Studies, University of Alabama at Birmingham, Birmingham, AL 35294, USA; 2 School of Communication, Florida State University, Tallahassee, FL 32306, USA; 3 Bob Schieffer College of Communication, Texas Christian University, Fort Worth, TX 76129, USA

**Keywords:** environmental health, social media, textual analysis, framing, air pollution

## Abstract

According to the latest report by the World Health Organization, air pollution, one of the planet’s most dangerous environmental carcinogens, has become one of the leading causes of cancer-related deaths. In China this is a particularly crucial issue, with more than 100 cities and close to one billion individuals threatened by haze due to heavy air pollution in recent years. Beyond traditional channels, the rise of social media has led to greater online haze-related information sharing. Formative research suggests that Weibo is playing a larger role in the process of information seeking than traditional media. Given the severity of haze and the influential role of Weibo, a textual analysis was conducted based on Sina Weibo (Chinese Twitter) to provide health decision-makers and media consumers knowledge on how environmental health issues such as haze are framed in Chinese social media. Framing theory served to explain the differences across various outlets: *People’s Daily*, *China Daily*, and the Chinese version of the *Wall Street Journal*. By analyzing 407 Weibo posts, five major frames emerged: (1) governmental concern, (2) public opinion and issue management, (3) contributing factors and effects, (4) socializing haze-related news, and (5) external haze-related news.

## 1. Introduction

According to the World Health Organization (WHO), over 7 million deaths, 1-in-8 global deaths, can be attributed to indoor and outdoor pollution, leading it to be considered one of the most dangerous environmental carcinogens [1,2]. Increasingly, China has been subject to serious outcomes of air pollution, with over 100 cities threatened by haze [3,4,5]. In the past decades, multiple Chinese cities have reported air pollution levels significantly higher than the health-based standards [6]. The health impacts associated with air pollution have become a growing concern in China [7]. This highlights the importance of understanding how air pollution-related information is portrayed and shared by media, especially the rapid-growing social media channels in China. Grounded in framing theory, this study utilized the textual analysis method to explore how information on air pollution is reported on Weibo, one of the largest social media platforms in China.

### 1.1. Environmental Threats: Air Pollution

Air pollution has been defined by WHO [1] as a “chemical, physical, or biological agent that modifies the natural characteristics of the atmosphere” (para. 1) and is categorized as indoor, household air pollution (HAP), or outdoor, ambient air pollution (AAP). Currently, HAP accounts for approximately 4.3 million deaths while the remaining deaths are due to AAP [3]. In 2012, Asia alone suffered over 2 million deaths due to causes associated with air pollution. These figures are nearly double previous measures, confirming what has long been suspected—air pollution is the world’s largest environmental health concern [8]. Studies indicate that the exposure levels of air pollution have increased significantly in some areas of the world, especially in rapidly industrializing countries with large populations [9]. These findings carry serious consequences, as WHO [8] has assessed air pollution to be one of the world’s leading causes of cancer alone with multiple other health conditions such as ischemic heart disease, stroke, and lung cancer. A 2016 national survey of energy consumption [10] in China found that heating accounts for about 50% of all residential energy usage in urban areas and in rural areas most households rely on “kangs” (solid fuel heating stoves), which are known for high pollutant emissions and smoke backflow [11]. The Global Burden of Disease project ranked HAP from cooking in China as the second most significant environmental risk factor for premature death [10].

Additionally, in China, due to rapidly expanding economic and industrial sectors, air pollution poses a serious environmental health concern. According to Chen et al. [11], China has some of the highest levels of particulate matter, specifically in megacities such as Beijing, Shanghai, and the Pearl River Delta region, with Guangzhou, Shenzhen, and Hong Kong having the highest rates. Potentially holding the world’s worst air pollution figures, China is now focused on mitigating the health risk posed by air pollution. Recently, epidemiological studies on air pollution have been conducted to examine the short-term and long-term health effects of air pollution on the Chinese population. Research indicates that an estimated 500 million Chinese individuals have shorten lifespans—5.5 years less on average—due to air pollution [11]. As of 2013, 1.4 million km^2^ of China was covered in haze, leading to serious consequential health concerns for 800 million Chinese people [12].

As one of the most life-threating environmental problems in China, some experts believe that haze has become more serious than the SARS epidemic [13]. In December 2013 more than 100 cities in China were threatened by haze, which endangered the health of more than one billion people, and was referred to as ‘airpocalypse’ by media. In 2013, haze in Beijing was so terrible that the Chinese government issued urgent warnings and closed four major highways, which prompted a frenzied buying of air filters and protective face masks [14]. During this time, haze permeated people’s indoor and outdoor spaces, reaching a pollution level 40 times that which is considered safe by WHO. According to the director of the Chinese Academy of Engineering and Guangzhou Institute of Respiratory Diseases, Zhong Nanshan, haze has greater carcinogenic effects than cigarette smoking [13].

Due to the severity of the environmental condition and serious effect of haze in China, the examination of media portrayals, or media framing, has emerged as an important area of research. With the growing importance of social media’s influence on consumers in China, it is critical for researchers to examine how Chinese news organizations adopt social media to deliver haze-related information. In the following section, the importance of examining the haze-related information on Weibo, one of the largest social media platforms in China, is discussed.

### 1.2. Social Media and Environmental Communication

Social media has been defined as a collection or group of Internet-based platforms that allow individuals to create, consume, and share content [15]. Through social media, users are able to engage with the creation and transference of information, along with the possibility of interacting with others [16]. To date, there are numerous social media platforms, such as social networking sites (e.g., Facebook), microblogging (e.g., Twitter), video sharing (e.g., YouTube), and others [15]. Globally, the usage of social media has grown at exponential rates, with China being one of the countries with the most users. Even despite the lack of access to platforms such as Facebook and Twitter, China usage rates remain high due to local social media sites specifically tailored for the Chinese market [17].

Weibo, one of most popular Chinese social media platforms, reached 462 million active accounts in December 2018, consisting of accounts from more than 37,000 media organizations, 130,000 corporations, and 170,000 government offices [18]. Weibo, sometimes described as a hybrid of Twitter and Facebook, combines elements of bulletin board systems (BBS), blogs, and social networking sites (SNSs). Weibo users are able to connect with other users by postings multimedia content (e.g., text, pictures, audios, videos), sending instant messages, ‘liking’ posts, and ‘following’ favorite accounts. Beyond being a popular platform for social interactions, much of its success is due to its essential role as the third largest source of information among Chinese Web users [19]. The authors of [20] argued that with their more than one billion users, Sina, Tencent, and other Weibo services help Chinese Internet users to receive and share information more efficiently than previous technologies. Liang [21] pointed out that with more than 46.3 million daily active users, Weibo is regarded as a critical platform in the dissemination of public information.

Weibo has become one of the major platforms for people in China to seek and scan health information [22]. In addition to Weibo’s power of rapidly delivering information and its influence on Chinese Internet users, it contains the ability to deliver rich text, which allows for adequate data collection to conduct textual analysis. Therefore, Weibo was chosen in this study as the social media platform to examine how health-related haze information is portrayed by different news organizations. To better analyze the haze-related information on Weibo, framing theory was adopted as the theoretical framework of this textual analysis.

### 1.3. Framing Theory

Framing has been defined as a process in which the message sender selects “…aspects of a perceived reality and make[s] them more salient in a communicating text (or visual), in such a way as to promote a particular problem definition, causal interpretation, moral evaluation, and/or treatment recommendation for the item described” [23] (p. 52). Entman [23] further stated that frames manifest in the presence or the omission of certain keywords, phrases, or images. Under the theoretical framework of framing, previous research has examined how Chinese and U.S. media has portrayed the same events from different perspectives in a variety of fields, such as politics, economics, social events, natural disasters, and public health. It has been found that Chinese and U.S. media tended to adopt different perspectives for the same event, and degrees of these differences vary according to the associated situations. Luther and Zhou [24] examined news frames in the newspaper coverage of SARS in China and the U.S. and found that although major news frames were present in both U.S. and Chinese news items, frames themselves varied with economic consequences, responsibility, and leadership. Moreover, conflict frames were more frequently emphasized in the U.S. newspapers. Similarly, Wu’s study [25] on HIV/AIDS news reports from the Xinhua News Agency of China and the Associated Press (AP) of the United States found that there was strong evidence of an anti-government stance in the AP’s report whereas the report by Xinhua had a strong pro-government valence. Three major themes—the dishonesty/oppression theme, the human rights abuser theme, and the incompetence theme—jointly supported the anti-government theme in AP’s report. In contrast, the defense theme, the progress theme, and the ambivalence/ambiguity theme were developed to support the pro-government theme in Xinhua’s report. Wu [25] concluded that news is “a socially constructed product, not an objective mirror of social reality” (p. 251).

With the rapid development of social media, traditional news platforms are losing their leading position in information delivery. Instead, social media, which functions distinctively different from traditional news platforms, is gaining a bigger role in influencing media consumers in China. Yet, limited studies have investigated whether the themes existing in Chinese newspapers still exist in the social media environment, especially in public health, health, and environment communication fields. Given the increased reliance on media outlets for health information sharing, it is critical to understand if, when, and how stories are framed. Although newspapers in China have high circulation rates, social media’s ability to disseminate news far exceeds standard mass media approaches (e.g., newspaper, magazine, television).

**RQ1:** Are haze-related news items represented within certain frames?

Previous studies have demonstrated that Chinese and the U.S. news agencies adopted different perspectives in their traditional news platforms, such as newspapers, yet whether the cross-country difference still exists in social media leads to the following research question.

**RQ2:** If framing differences exists, what are the major news frames for haze-related news and how might these be differentiated by outlet/source?

## 2. Materials and Methods

A textual analysis was conducted to investigate the potential themes present in the Weibo posts of Chinese news agencies and their potential differences when compared with the U.S. news agencies in the Chinese social media platform. Textual analysis is defined as a rigorous, in-depth, and systematic analysis and interpretation of textual material that allows for the emergence of patterns and themes [26]. Following the guide of Berg and Lune [26] for textural analysis, three sources (*People’s Daily*, *China Daily*, and the Chinese version of the *Wall Street Journal* on the Sina Weibo platform) were selected. In order to gain a more holistic understanding of how haze-related news are presented, it was important to have representation of three levels: high governmental affiliation, mid-level governmental affiliation, and low-level governmental affiliation. Governmental affiliation was determined by examining the sources website.

### 2.1. Data Sources

*People’s Daily* is the official newspaper of the Chinese government, which provides official and direct information on the policies and viewpoints of the government. *People’s Daily* is a daily newspaper published globally with a circulation rate of around 4 million [27]. Besides the main edition in Chinese, *People’s Daily* contains editions in English, French, Spanish, Japanese, and many other languages, and has been selected by the United Nations Educational, Scientific, and Cultural Organization as one of the most authoritative and influential newspapers in China [28]. *People’s Daily* was selected to represent a Chinese news agency due to its high governmental association and the fact that its Weibo account has close to 20 million followers and over 25,000 posts.

Established in 1981 as the only English language newspaper at that time, *China Daily* was developed to introduce international information to Chinese people as well as send China’s messages to the world as part of the country’s economic reforms and open-door policy. Belonging to the group of international newspapers in China, *China Daily* serves as an example of the Chinese government’s effort to gain the international community’s attention. *China Daily* operates “under the same rules as the local language press and allows outsiders a glimpse of the country and its perspective on the world” [29] (p. 156). With the development of the market economy, *China Daily* is now under the influence of both the Chinese government’s censorship as well as the commercialization effect of the market. Thus, *China Daily* was selected as a representative news agency with a moderate level of association with the Chinese government. The Weibo account of *China Daily* contains over 2 million followers over 27,000 posts.

The *Wall Street Journal (WSJ)* is a daily U.S. newspaper with a special emphasis on business and economic news. With a circulation rate of around 2.4 million, *WSJ* is the largest newspaper in the U.S. In China, the Chinese version of the *WSJ* website was established in 2002 for the purpose of providing high-quality economic news and deep analysis of global Chinese markets. As a Chinese-language online publication of Dow Jones & Company, the Chinese version of *WSJ* is a branch of the English-language *WSJ*, and shares similar, if not the same, information. The Chinese version of *WSJ* was selected as an example of a U.S. news agency that has a relatively low level of association with the Chinese government. The Weibo account of the Chinese version of *WSJ* has around 2.7 million followers and over 73,000 posts.

### 2.2. Data Collection

For this study, data were retrieved from the Sina Weibo (www.Weibo.com) accounts of *People’s Daily*, *China Daily*, and *WSJ*. Like Twitter, Weibo provides a user verification service for the purposes of verifying and authorizing users’ identities, including both individual accounts and organizational accounts. Verified organizational users are labeled with blue “V” signs while verified individual users are labeled with golden “V” signs. The data for this study were all collected from the three verified Weibo accounts of *People’s Daily*, *China Daily*, and *WSJ* with blue “V” signs. The data were collected by employing a time series approach. Over a period of 6 months, posts related to haze were retrieved from the three accounts and analyzed for characteristics. The search engine provided by Sina Weibo was adopted to locate the related information with the keyword “haze.” A total of 407 posts were found, with 254 from *People’s Daily*, 47 from *China Daily*, and 106 from the Chinese version of *WSJ*. Each post was treated as a basic unit of analysis and classified based on its themes.

### 2.3. Coding Procedure

Following Strauss and Corbin’s [30] constant comparative method, emergent themes (codes) were analyzed systematically and categories were consolidated where appropriate. Each post could be coded as multiple categories, and codes became the basis for analysis. Two native Chinese-speaking coders received coding training from the primary investigator for the study. Framing theory was used as the theoretical guidance, and the frames identified in the following section were the significant themes that emerged during the coding procedure. Once the initial coding scheme was developed, sample sections from the three Weibo accounts that fell outside of the employed data collection time frame were coded by each author and agreement was reached by consensus on points of disagreement.

## 3. Results

By analyzing the 407 Weibo posts on haze-related information from the three news agencies’ social media accounts, five major frames emerged. These include: (1) governmental concern, (2) public opinion and issue management, (3) contributing factors and effects, (4) socializing haze-related news, and (5) external haze-related news. In the following sections, each theme is described in detail.

### 3.1. Governmental Concern

All three of the news agencies’ social media accounts greatly emphasized the Chinese government’s strong emphasis on haze and the corresponding policy adjustments for haze management, especially during the conference session of the National People’s Congress and the Chinese Political Consultative Conference. *People’s Daily* contained the most posts on the governments’ efforts to manage haze. For instance, one post from *People’s Daily* provided information shared by the Prime Minister in regard to the importance of combating haze through the implementation of PM2.5, atmospheric particulate matter (PM) with a diameter of less than 2.5 micrometers, monitoring assessments.

We need to fight the haze by changing our way of producing goods and life style chocies. In the past year, we have enacted 10 measures for managing the air pollution, and set numerous new monitoring sites of PM2.5 in 161 cities, which means China has the most PM2.5 monitoring sites among developing countries.

### 3.2. Public Opinion and Issue Management

All three of the news agencies provided information on critical thinking and viewpoints on haze management for both the government and the public with information sources of editorial views from newspapers, blogs from the public, and suggestions from social media account managers. For the public, most of the suggestions focused on discouraging people from the consumption of fireworks and firecrackers during the Chinese Spring Festival. One post from *China Daily* wrote:

The coverage of haze in the North China does not discourage people’s enthusiasm for fireworks. The deafening sounds of fireworks constantly remind us our inability to “defend our air”, even though we always talk about our responsibility for the haze. Protecting the environment is everybody’s responsibility.

Most of the posts shared a viewpoint that was critical of the government’s development model, which is believed to be sacrificing the environment to the rapid development of economics, and suggested “China should slow down its pace of economic development and wait for the environment to recover.” One post from *People’s Daily* argued: “Although the management of haze could not happen in one night, the speed of managing should not always be behind the speed of deterioration. We need to re-think the meaning of rapid economic development if it means sacrificing the environment we live in. We need real action to manage the haze!”

### 3.3. Contributing Factors and Effects of Haze

Although all of the news agencies contained posts on the possible reasons causing haze, *People’s Daily* tended to deliver the most information on the potential causes. For instance, one of posts by *People’s Daily* demonstrated that scientists have determined the six major sources of haze in Beijing. Another post explained the high prevalence of haze in Eastern China. In addition, some other posts stated the links between the quality of gasoline used in China and the generation of haze.

The potential effects of haze have been analyzed from many aspects, such as the harmful effects on human health, the heavy burden on public transportation, and the stimulation of relative industries’ current and future developments. For instance, a post from *China Daily* discussed the potential link between haze and mental health. Another post from the Chinese version of *WSJ* provided opinions on whether the haze could cause cancer. Posts on the effects of haze from *People’s Daily* were mostly about the great burden on traffic due to heavy haze. The Chinese version of *WSJ* paid more attention to the haze’s economic effects, such as the booming development of industries producing haze protection products and the long-term development of green automotive industries.

The forecast of upcoming haze was another major focus of news agencies, particularly the *People’s Daily*. Both *China Daily* and the Chinese version of *WSJ* tended to provide only information without any suggestions for protection; meanwhile, *People’s Daily* more frequently added tips on haze protection at the end of the forecast. For instance, one post from *China Daily* stated: “Beijing and Tianjin, as well as parts of Hebei and Shandong provinces expect ‘moderate’ air pollution from Wednesday to Thursday morning, heavy in some places.” With similar information, the post by *People’s Daily* added one more sentence: “Remember to bring a respirator with you when walking outside.”

Haze-related science facts were found to be another important news item that agencies posted on their social media accounts. For instance, one of the posts by *China Daily* explained the reasons why light rain might contribute to an increase in haze. Another post highlighted the “popularization” of the scientific concepts behind haze; *People’s Daily* introduced the components of PM2.5, the potential harmful effects of particulate matter, and the prevention measures that could be taken.

### 3.4. Socializing Haze-Related News

Several pieces on haze-related social news reported in newspapers were also posted in the news agencies’ social media accounts. Some of the stories were highly related to haze, while others only mentioned the term “haze” once throughout the whole article. The posts from *China Daily* and the Chinese version of *WSJ* demonstrated greater interest in reporting the struggles of foreigners living in Beijing and Shanghai and the major influence that haze had on their lives. Several posts told stories of foreigners’ decisions to leave China mainly due to the unbearable air quality. One example was a report from *China Daily* depicting the story of a company executive with a graduate degree deciding to resign from his high-level position in the company in order to join the Shaolin Temple, where he would be less affected by haze. In the article, the man mentioned that he was the envy of many of his colleagues in the Shaolin Temple because they were ‘safer’ from the haze.

All of the news agencies posted haze pictures with simple explanations of the presenting conditions in the pictures. All of the pictures provided by *China Daily* and the Chinese version of *WSJ* were about heavy haze, with blurred scenes and people wearing respirators. Conversely, some of the pictures from *People’s Daily* contained clear skies, green grass, and colorful flowers, with explanations like “Although we are suffering from the haze now, please do not forget the beauty of nature. Hope we will see it again very soon.”

### 3.5. External Haze-Related News

The Chinese version of *WSJ* and *People’s Daily* were found to repost news reports on haze occurring in other countries. For instance, *People’s Daily* posted one report on its social media account about the haze happening in Los Angeles, and the Chinese version of *WSJ* contained several posts on the haze situation in Singapore. One post in the Chinese version of *WSJ* wrote:

Singapore is suffering the worst haze in its history. The haze covering Singapore became worse on Wednesday, exceeding the highest harmful level in Singapore’s recorded history.

Besides the shared themes listed above, there were several themes uniquely contained in the social media accounts of certain news agencies. The differentiating quality of the Chinese version of *WSJ* was that it provided comparative information from both the Chinese government and the United States office in China. One example was the number of days that haze was reported to have occurred in China from 2008 to 2014. By providing information from both sources, a clear difference appeared, with the data from the Chinese government revealing a dramatically smaller number of haze days in the past 7 years. Besides this, the Chinese version of *WSJ* provided haze-related information in more diverse ways, such as videos. Additionally, it reported haze in a less serious manner, such as a report on the need for Chinese commercial pilots to practice blind landings due to the frequency of haze conditions. Haze-related information from foreign sources has also been provided to readers by *WSJ*. The advantage afforded by social media in interacting with their audience has been highly employed by the Chinese version of *WSJ*. For instance, the Chinese version of *WSJ* used the vote function and encouraged audiences to provide their opinions on haze-related issues by voting. *People’s Daily* also encouraged its audience to become involved in discussion on this topic. During one exchange, *People’s Daily* used its social media account with the sole purpose of inspiring its audience to provide tips on how to manage haze. Overall, *People’s Daily* showed a strong tendency to adopt positive and persuasive messages on haze-related issues. As mentioned above, instead of providing pictures of haze with a negative valence, *People’s Daily* provided positive images and encouraged the audience to wait patiently for the upcoming beauty of nature. *People’s Daily* also provided persuasive messages that suggested that in the future there would be substantial improvement to the environment. Compared to the other two news agencies, *China Daily* showed its distinctiveness through the usage of less formal language and pictorials, specifically cartoons. One example was a cartoon in which Santa Claus hit a tree due to the heavy haze.

## 4. Discussion

The emergent themes found among the three news agencies demonstrate the commonality of certain themes in reporting haze-related issues, such as the criticism of the Chinese government’s policies for economic development by sacrificing the environment, and suggestions that everyone has a responsibility to take care of the environment. However, the different news agencies held varying levels of criticism toward the government, with the lowest level of criticism from *People’s Daily* and the highest level of criticism from the Chinese version of *WSJ*. The Chinese version of *WSJ* went as far as to distrust information provided by the Chinese government, and offered comparative information from U.S. media sources. The Chinese version of *WSJ* stands out as the news agency in Chinese social media with the most diverse form of information delivery mechanisms, the inclusion of various news sources, and the provision of critical inquiry on the quality of information originating from the Chinese government.

*China Daily’s* social media account contained the smallest number of only haze-related posts among all three news agencies. Though it contained a similar number of total posts as *People’s Daily*, *China Daily* showed comparatively less attention to the topic of haze. The reason behind this phenomenon could be explained by the understanding that *China Daily* aims to provide information about China to the world, and under the censorship of Chinese government, *China Daily* should not provide a great deal of negative information about China as it may harm China’s reputation. In general, the issue of haze is one of the topics that holds perhaps the greatest potential of harming China’s international image. Therefore, sources such as *China Daily* would be far less inclined to mention the harmful effects of haze. Similarly, *People’s Daily* is the news organization directly associated with the Chinese government, so it mostly serves the government’s purposes. The positive perspectives of haze-related issues and persuasive messages on a better future could serve as placebos to maintain social stability, which is one of the duties of the Chinese government.

## 5. Conclusions

Air pollution is a serious environmental issue that significantly influences the health of Chinese people. To understand how haze-related information is framed in Chinese social media, a textual analysis was conducted based on information posted on Sina Weibo by three different media sources: *People’s Daily*, *China Daily*, and the Chinese version of the *Wall Street Journal.* After analyzing 407 Weibo posts based on framing theory, we identified five major frames: (1) governmental concern, (2) public opinion and issue management, (3) contributing factors and effects, (4) socializing haze-related news, and (5) external haze-related news. The findings of this study provide health decision-makers and media consumers with information on the framing of haze in Chinese social media.
